# The Risk of Peripheral Arterial Disease after Parathyroidectomy in Patients with End-Stage Renal Disease

**DOI:** 10.1371/journal.pone.0156863

**Published:** 2016-06-10

**Authors:** Yueh-Han Hsu, Hui-Yi Yu, Hsuan-Ju Chen, Tsai-Chung Li, Chih-Cheng Hsu, Chia-Hung Kao

**Affiliations:** 1 Department of Health Services Administration, China Medical University, Taichung, Taiwan; 2 Division of Nephrology, Department of Internal Medicine, Ditmanson Medical Foundation Chia-Yi Christian Hospital, Chia-Yi, Taiwan; 3 Department of Nursing, Min-Hwei Junior College of Health Care Management, Tainan, Taiwan; 4 Division of Endocrinology and Metabolism, Department of Internal Medicine, Ditmanson Medical Foundation Chia-Yi Christian Hospital, Chia-Yiy, Taiwan; 5 Department of Sports Management, Chia Nan University of Pharmacy and Science, Tainan, Taiwan; 6 Management Office for Health Data, China Medical University Hospital, Taichung, Taiwan; 7 College of Medicine, China Medical University, Taichung, Taiwan; 8 Graduate Institute of Biostatistics, College of Public Health, China Medical University, Taichung, Taiwan; 9 Department of Healthcare Administration, College of Health Science, Asia University, Taichung, Taiwan; 10 Institute of Population Health Sciences, National Health Research Institutes, Zhunan, Miaoli, Taiwan; 11 Graduate Institute of Clinical Medical Science and School of Medicine, College of Medicine, China Medical University, Taichung, Taiwan; 12 Department of Nuclear Medicine and PET Center, China Medical University Hospital, Taichung, Taiwan; Emory University, UNITED STATES

## Abstract

**Purpose:**

The changes of the risk of peripheral arterial disease (PAD) in patients with end-stage renal disease after parathyroidectomy are scant.

**Methods:**

We used a nationwide health insurance claims database to select all dialysis-dependent patients with end-stage renal disease aged 18 years and older for the study population in 2000 to 2006. Of the patients with end-stage renal disease, we selected 947 patients who had undergone parathyroidectomy as the parathyroidectomy group and frequency matched 3746 patients with end-stage renal disease by sex, age, years since the disease diagnosis, and the year of index date as the non-parathyroidectomy group. We used a multivariate Cox proportional hazards regression analysis with the use of a robust sandwich covariance matrix estimate, accounting for the intra-cluster dependence of hospitals or clinics, to measure the risk of peripheral arterial disease for the parathyroidectomy group compared with the non-parathyroidectomy group after adjusting for sex, age, premium-based income, urbanization, and comorbidity.

**Results:**

The mean post-op follow-up periods were 5.08 and 4.52 years for the parathyroidectomy and non-parathyroidectomy groups, respectively; the incidence density rate of PAD in the PTX group was 12.26 per 1000 person-years, significantly lower than the data in the non-PTX group (24.09 per 1000 person-years, adjusted HR = 0.66, 95% CI = 0.46–0.94).

**Conclusion:**

Parathyroidectomy is associated with reduced risk of peripheral arterial disease in patients with end-stage renal disease complicated with severe secondary hyperparathyroidism.

## Introduction

The risk of peripheral arterial disease (PAD) in patients with end-stage renal disease (ESRD) is 10 times higher than non-ESRD patients In the United States [1]. The prevalence of PAD in ESRD patients is 17–48% [2, 3]. PAD, as reviewed by O’Hare et al, confers substantial risks for morbidity and mortality in the ESRD population [2]. From previous studies, risk factors of PAD in general population and ESRD might be different. Reported risk factors for PAD in general population including age, male, smoking, diabetes mellitus (DM), chronic kidney disease (CKD), albuminuria and hepatitis C [4–9]. DM is still an important risk factor of PAD in ESRD [10]; while secondary hyperparathyroidism (SHPT) was reported to be a critical risk factor of PAD in ESRD [10].

SHPT was traditionally considered as an important factor for cardiovascular morbidity and mortality of ESRD patients [11]. Though certain previous studies reported negative associations between PTH levels and CV morbidity [12, 13], it was considered that low PTH levels represented residual confounding by nutritional status [14]. Parathyroidectomy (PTX) is the main treatment for severe SHP refractory to medical treatment. We previously reported PTX to be associated with 43% lower risk of stroke [15]. Several studies reported reduced overall mortality and cardiovascular mortality in SHPT patients who received PTX [16–19]. However, research addressing CV morbidity statuses in these patients is few. In the report by Conzo et al, PTX did not modify CV morbidity and mortality rates in hemodialysis (HD) patients with SHPT [20]. Ishani et al reported PTX to be associated with significant morbidity in the 30 days after hospital discharge and in the year after the procedure, emphasizing evidence-based determinations for the indication for PTX [21].

The relationship between PTX and incident PAD in dialysis-dependent ESRD patients was seldom approached. The aim of this research was to investigate this relationship in a retrospective cohort by using a nationwide health insurance database. We hypothesized that receiving PTX might be associated with a reduced risk of incident PAD in ESRD patient

## Methods

### Data source

The Taiwan National Health Insurance (NHI) program has offered comprehensive, universal health insurance to all residents of Taiwan since 1995 and covers more than 99% of the residents. The National Health Insurance Research Database (NHIRD) is a research database developed and managed by National Health Research Institute (NHRI), and confidentiality is maintained according to the directives of the Bureau of NHI. In this study, we used the Registry of Catastrophic Illnesses Patient Database (RCIPD), which is part of the NHIRD. The RCIPD contains health claims data for the treatment of catastrophic illness and includes 30 categories of diseases requiring long-term care. For privacy protection, all insured subjects had been scrambled cryptographically to attain anonymity. The diagnoses and procedures are coded in the International Classification of Disease, Ninth Revision, Clinical Modification (ICD-9-CM) format.

### Study population

**Fig 1:**
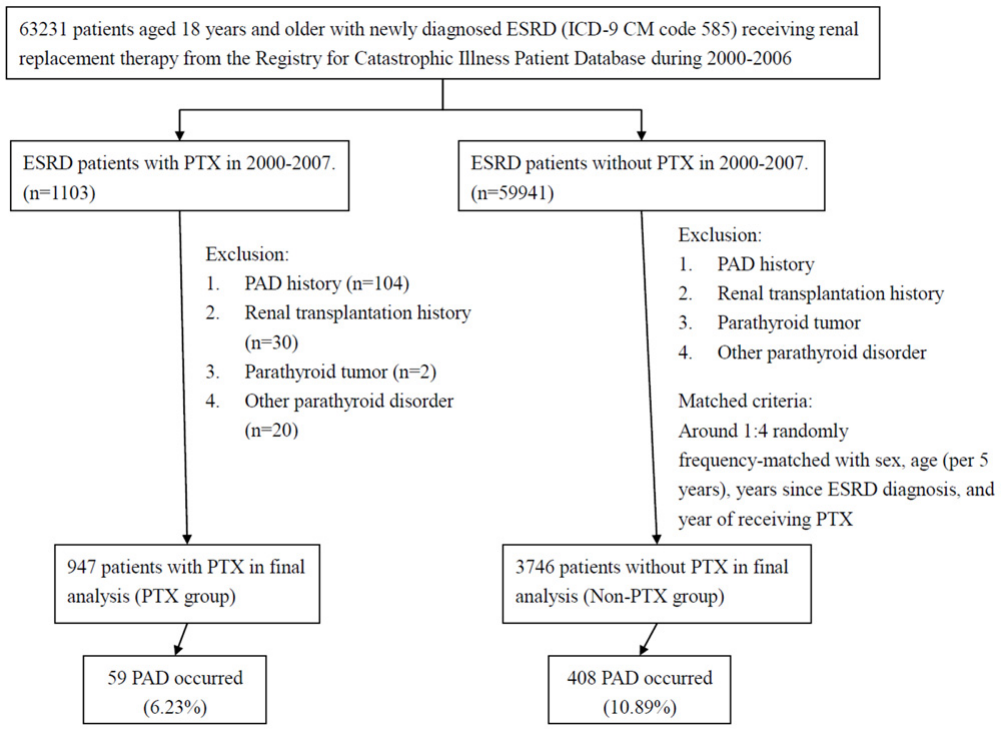
Flow chart showing selection of study subjects. Abbreviation: ESRD, end stage renal disease; PAD, peripheral arterial disease; PTX, parathyroidectomy.

Fig 1 shows the study framework. From 2000 to 2006, we selected ESRD patients (aged ≥ 18 years) defined as those who had catastrophic illness registration cards for ESRD (ICD-9-CM 585) and who underwent long-term renal replacement therapy. From the ESRD population, we conducted a population-based retrospective cohort study among patients who newly received PTX (ICD-9 codes for procedure 06.8) but without a history of PAD (ICD-9-CM 440.2, 440.3, 440.8, 440.9, 443, 444.22, 444.8, 447.8, and 447.9), renal transplantation (ICD-9-CM V42.0), parathyroid tumor (ICD-9-CM 194.1 and 227.1), or other parathyroid disorder (ICD-9-CM 252.8) during 2000–2007. The diagnosis of PAD was made clinically. The date of first-time PTX was defined as the index date. For each ESRD patient who received PTX, we selected 4 patients randomly from the remaining ESRD patients without receiving PTX and matched on sex, 5-year age interval, dialysis vintage (years since ESRD diagnosis and receiving regular dialysis), and year of index date, using the inclusion criteria similar to the PTX group.

The demographic factors included sex, age (in age group of 18–34 years, 35–49 years, 50–64 years, and 65 years and above), insured amount, and urbanization. Amount of insurance premium was categorized into three levels: <15000, 15000–29999, and ≥30000 New Taiwan (NT) dollars per month. The insurance premium amount of an individual was determined by his/her work salary. Urbanization level was defined according to the NHRI report [22]. Level 1 indicates the most urbanized area and level 4 indicates the least urbanized area.

The baseline comorbidity history was determined for each patient; these comorbidities included diabetes (ICD-9-CM 250), hyperlipidemia (ICD-9-CM 272), hypertension (ICD-9-CM 401–405), atrial fibrillation (AF, ICD-9-CM 427.31), congestive heart failure (CHF, ICD-9-CM 398.91, 425, and 428), ischemic heart disease (IHD, ICD-9-CM 410–414), stroke (ICD-9 CM 430–438), chronic obstructive pulmonary disease (COPD, ICD-9-CM 491–494 and 496), obesity (ICD-9-CM 278), and alcohol-related disease (ICD-9-CM 291, 303, 305, 571.0, 571.1, 571.2, 571.3, 790.3, and V11.3).

The interesting outcome was newly diagnosed PAD (ICD-9-CM 440.2, 440.3, 440.8, 440.9, 443, 444.22, 444.8, 447.8, and 447.9) recorded in the NHIRD. Patients were followed from the index date to the date on which any of the following first occurred: the date of PAD diagnosis, date of withdrawal from the NHI program, death, or 31 December 2011. We focused on the changes of risk of PAD at a nationwide level; changes in individual items were out of the realm of the study.

### Ethics Statement

The NHIRD encrypts patient personal information to protect privacy and provides researchers with anonymous identification numbers associated with relevant claims information, including sex, date of birth, medical services received, and prescriptions. Therefore, patient consent is not required to access the NHIRD. This study was approved to fulfill the condition for exemption by the Institutional Review Board (IRB) of China Medical University (CMUH104-REC2-115). The IRB also specifically waived the consent requirement.

### Statistical analysis

Distribution of sex, age, insured amount, urbanization, and comorbidities were compared between the PTX and the non-PTX groups. To consider the dependence of clustered data, differences were examined using the Cochran-Mantel-Haenszel (CMH) chi-squared test for the categorical variables and linear mixed model for the continuous variables. The sex-, age-, comorbidity-specific incidence density of PAD (per 1000 person-years) was calculated each group. Kaplan–Meier analysis was used to plot the cumulative incidence of PAD and a log-rank test was used to test the differences between the two groups. Univariable and multivariable Cox proportional hazard regression models with the use of a robust sandwich covariance matrix estimate that accounts for the intra-cluster dependence of hospitals or clinics, were used to assess the risk of PAD and PAD-associated risk factors, and the models were adjusted for sex, age, insured amount, urbanization, and comorbidity. We also performed sex-, age-, and comorbidity-stratified analysis to investigate the association between PTX and PAD. Hazard ratios (HRs) and 95% confidence intervals (CIs) were calculated to quantify the risk of PAD. SAS version 9.3 (SAS Institute, Cary, NC, USA) was used for the data analyses; two-sided tests were performed, and p-value<0.05 was considered statistically significant.

## Results

This study included a cohort containing 947 ESRD patients with newly receiving PTX (the PTX group) and 3746 patients without receiving PTX (the non-PTX group). The mean age and corresponding standard deviation (SD) of the PTX group was 50.90 years (11.94 years), and women were predominant (66.10%). Compared with the non-PTX group, the PTX group exhibited a lower prevalence of low income, DM, and stroke, and higher prevalence of hypertension and obesity (Table 1).

**Table 1:** Demographic factors and comorbidity of patients with end stage renal disease according to PTX status.

	Non-PTX N = 3746		PTX N = 947		
Variables	n	%	n	%	p-value
**Sex**					0.98
Women	2478	66.15	626	66.10	
Men	1268	33.85	321	33.90	
**Age at receiving PTX, years**					0.99
18–34	357	9.53	98	10.35	
35–49	1333	35.58	335	35.37	
50–64	1584	42.29	396	41.82	
≥65	472	12.60	118	12.46	
Mean (SD)	51.24	(11.87)	50.90	(11.94)	0.53
**Insured amount (NT$/ month)**					<0.001
<15000	1868	49.87	432	45.62	
15000–29999	1419	37.88	350	36.96	
≥30000	459	12.25	165	17.42	
**Urbanization**					0.93
Level 1 (highest)	1005	26.83	259	27.35	
Level 2	1252	33.42	294	31.05	
Level 3	654	17.46	190	20.06	
Level 4 (lowest)	835	22.29	204	21.54	
**Comorbidity**					
Diabetes	1325	35.37	153	16.16	<0.001
Hyperlipidemia	1734	46.29	439	46.36	0.70
Hypertension	3369	89.94	877	92.61	0.002
AF	85	2.27	17	1.80	0.32
CHF	1138	30.38	245	25.87	0.16
IHD	1588	42.39	380	40.13	0.30
Stroke	446	11.91	60	6.34	<0.001
COPD	892	23.81	213	22.49	0.80
Obesity	27	0.72	14	1.48	0.006
Alcohol-related disease	85	2.27	13	1.37	0.40
**Follow-up time, year, mean (SD)**	4.52	(2.17)	5.08	(1.88)	

Abbreviation: PTX, parathyroidectomy; SD, standard deviation; AF, atrial fibrillation; CHF, congestive heart failure; IHD, ischemic heart disease; COPD, chronic obstructive pulmonary disease.

**Fig 2:**
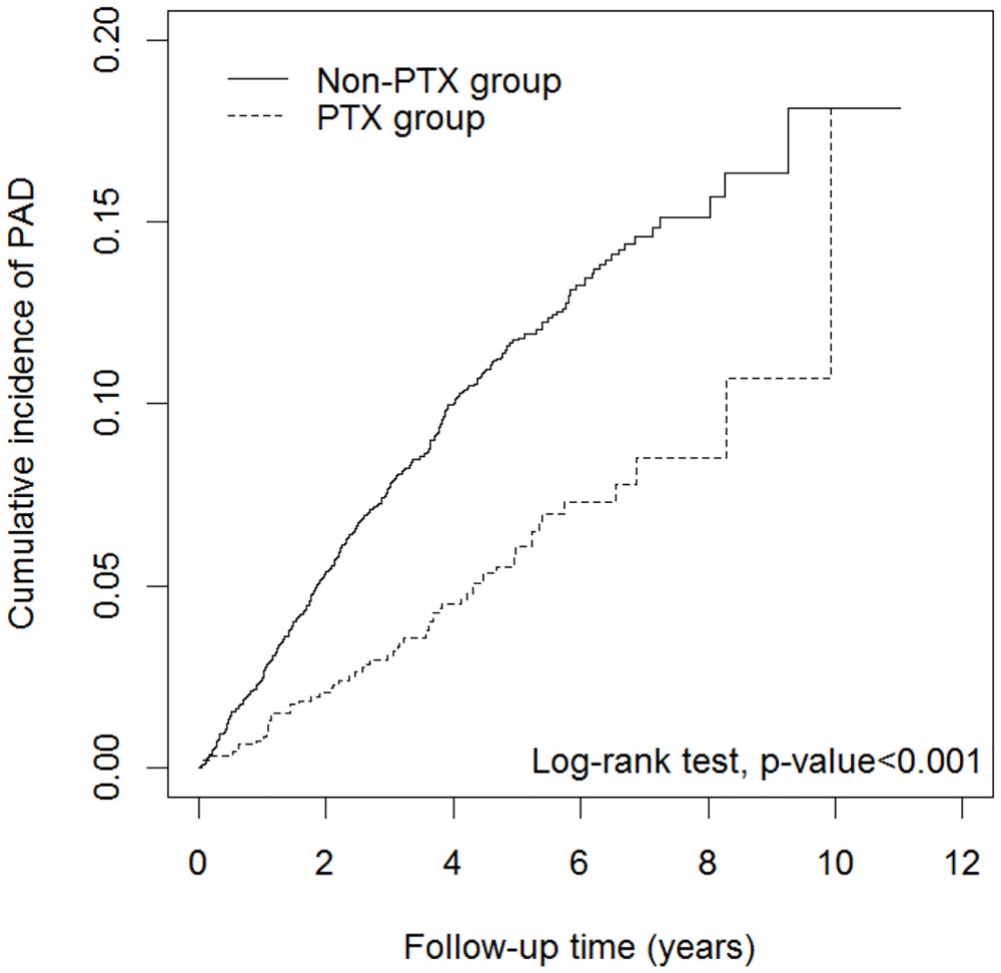
Cumulative incidence curves of PAD for PTX and non-PTX groups. Abbreviation: PAD, peripheral arterial disease; PTX, parathyroidectomy.

During an average follow-up of 4.63 years, 59 patients in the PTX group and 408 patients in the non-PTX group developed PAD. The incidence density of PAD in the PTX group was 12.26 per 1000 person-years, significantly lower than the data in the non-PTX group (24.09 per 1000 person-years), with an adjusted HR of 0.66 (95% CI = 0.46–0.94; Table 2). Fig 2 shows that the cumulative incidence of PAD was significantly lower in the PTX group than the non-PTX group (log-rank test, p-value < 0.001).

**Table 2:** Hazard ratios (HRs) and 95% confidence interval of PAD associated with PTX and covariates.

Variables	Event no.	IR	Unadjusted HR (95%CI)	Adjusted HR^†^ (95% CI)
**PTX**				
No	408	24.09	1.00	1.00
Yes	59	12.26	0.51 (0.36–0.0.72)***	0.66 (0.46–0.94)*
**Sex**				
Women	306	21.14	1.00	1.00
Men	161	22.12	1.04 (0.87–1.25)	1.24 (1.01–1.53)*
**Age at receiving PTX, years**				
18–34	17	6.77	1.00	1.00
35–49	121	14.14	2.09 (1.14–3.81)*	1.67 (0.92–3.04)
50–64	250	29.18	4.24 (2.39–7.52)***	2.16 (1.17–4.01)*
≥65	79	37.30	5.37 (2.89–9.98)***	2.40 (1.22–4.70)*
**Insured amount (NT$/ month)**				
<15000	268	26.00	1.00	1.00
15000–29999	167	20.29	0.79 (0.64–0.99)*	1.03 (0.82–1.28)
≥30000	32	9.96	0.39 (0.28–0.53)***	0.55 (0.40–0.76)***
**Urbanization**				
Level 1 (highest)	127	21.54	1.00	1.00
Level 2	156	21.57	1.00 (0.75–1.34)	1.00 (0.73–1.37)
Level 3	91	23.54	1.09 (0.80–1.49)	1.05 (0.75–1.49)
Level 4 (lowest)	93	19.55	0.91 (0.68–1.22)	0.87 (0.64–1.17)
**Comorbidity**				
**Diabetes**				
No	174	10.73	1.00	1.00
Yes	293	52.87	4.87 (3.97–5.99)***	2.97 (2.32–3.80)***
**Hyperlipidemia**				
No	168	13.49	1.00	1.00
Yes	299	32.17	2.36 (1.91–2.90)***	1.28 (1.03–1.59)*
**Hypertension**				
No	16	6.48	1.00	1.00
Yes	451	23.39	3.54 (2.07–6.03)***	1.72 (1.00–2.97)
**AF**				
No	458	21.42	1.00	1.00
Yes	9	24.60	1.13 (0.56–2.28)	0.69 (0.33–1.45)
**CHF**				
No	268	16.63	1.00	1.00
Yes	199	35.28	2.09 (1.78–2.45)***	1.33 (1.13–1.57)***
**IHD**				
No	169	12.44	1.00	1.00
Yes	298	36.49	2.90 (2.40–3.50)***	1.66 (1.35–2.06)***
**Stroke**				
No	379	19.05	1.00	1.00
Yes	88	47.38	2.45 (1.98–3.03)***	1.26 (1.00–1.58)
**COPD**				
No	338	19.71	1.00	1.00
Yes	129	28.01	1.40 (1.09–1.80)**	0.96 (0.74–1.25)
**Obesity**				
No	462	21.41	1.00	1.00
Yes	5	28.04	1.30 (0.59–2.88)	1.12 (0.49–2.59)
**Alcohol-related disease**				
No	461	21.60	1.00	1.00
Yes	6	14.56	0.67 (0.29–1.53)	0.66 (0.29–1.49)

Abbreviation: PTX, parathyroidectomy; IR, incidence density, per 1,000 person-years; HR, hazard ratio; CI, confidence interval; AF, atrial fibrillation; CHF, congestive heart failure; IHD, ischemic heart disease; COPD, chronic obstructive pulmonary disease.

^†^ Multivariable analysis including PTX, sex, age(categorical), insured amount, urbanization, diabetes, hyperlipidemia, hypertension, atrial fibrillation, congestive heart failure, stroke, chronic obstructive pulmonary disease, obesity, and alcohol-related disease.

* p<0.05,

** p<0.01,

*** p<0.001.

Compared with younger patients (age 18–34), the risk of developing PAD is 2.16-fold (95% CI = 1.17–4.01) higher in those aged 50–64 years and 2.40-fold (95% CI = 1.22–4.70) higher in those aged 65 years and above; thus, the risk of developing PAD increases with age. Multivariate Cox proportional hazard analysis showed that PAD was independently associated with men (adjusted HR = 1.24, 95% CI = 1.01–1.53), diabetes (adjusted HR = 2.97, 95% CI = 2.32–3.80), hyperlipidemia (adjusted HR = 1.28, 95% CI = 1.03–1.59), CHF (adjusted HR = 1.33, 95% CI = 1.13–1.57), and IHD (adjusted HR = 1.66, 95% CI = 1.35–2.06).

Sex-specific analysis showed the incidence density rates of PAD in women and men with receiving PTX were 10.42 and 15.79 per 1000 person-years, respectively; lower than in the non-PTX group (24.15 and 23.97 per 1000 person-years, respectively). In addition, women showed 0.60-fold (adjusted HR = 0.60, 95% CI = 0.40–0.90) lower risk of PAD in the PTX group than the non-PTX group. Age-specific analysis showed that the PTX group had a lower risk of PAD compared to the non-PTX group in age group of 65 years and older (adjusted HR = 0.44, 95% CI = 0.23–0.84). Comorbidity-specific analysis showed that patients in the PTX group with comorbidity had a decreased risk of PAD compared with patients in the non-PTX group and with comorbidity (adjusted HR = 0.49, 95% CI = 0.35–0.70; Table 3).

**Table 3:** Incidence density and hazard ratios of PAD according to PTX status stratified by sex, age, and comorbidity.

	PTX						Compared to no PTX group	
	No			Yes			HR (95% CI)	
Variables	Event no.	Person-years	IR	Event no.	Person-years	IR	Unadjusted	Adjusted^‡^
**Sex**								
Women	273	11306	24.15	33	3167	10.42	0.43 (0.29–0.66)***	0.60 (0.40–0.90)*
Men	135	5632	23.97	26	1647	15.79	0.66 (0.42–1.03)	0.75 (0.46–1.22)
**Age at receiving PTX, years**								
18–64	337	15345	21.96	51	4289	11.89	0.54 (0.37–0.81)**	0.73 (0.49–1.08)
≥65	71	1593	44.58	8	525	15.23	0.33 (0.17–0.64)***	0.44 (0.23–0.84)*
**Comorbidity**^†^								
No	8	1119	7.15	1	187	5.35	0.76 (0.09–6.16)	0.79 (0.10–6.16)
Yes	400	15819	25.29	58	4627	12.53	0.50 (0.35–0.71)***	0.49 (0.35–0.70)***

Abbreviation: PTX, parathyroidectomy; IR, incidence density, per 1,000 person-years; HR, hazard ratio; CI, confidence interval.

^†^ Patients with any one of diabetes, hyperlipidemia, hypertension, atrial fibrillation, congestive heart failure, ischemic heart disease, stroke, chronic obstructive pulmonary disease, obesity, and alcohol-related disease were classified as the comorbidity group.

^‡^ Mutually adjusting for sex, age (continuous), insured amount, urbanization, and comorbidity.

* p<0.05,

** p<0.01,

*** p<0.001.

## Discussion

In this study, we observed that in ESRD patients who conducted PTX, there is a 34% risk reduction for incident PAD. Female sex and the presence of comorbidity are the two characteristics of the PTX group who might benefit from receiving the treatment.

SHPT is one of the major problems among long-term dialysis patients and is associated with increased vascular calcification, CV risk, and mortality [23–25]. Elevated PTH level predicts a greater likelihood of prevalent and incident CV events, including myocardial infarction (MI), stroke, and CV death [25, 26]. O’Hare et al reported that in dialysis patients, PAD was positively associated with the duration of dialysis and malnutrition status and was negatively associated with serum albumin and parathyroid hormone [10]. Because PAD has similar risk factors to CV disease, people with PAD might have other atherosclerotic disease concomitantly [27]. Besides, several studies found an association of PAD with CV mortality and morbidity [28, 29]. If PTX is associated with reduced CV mortality, one may expect that the risk of PAD be reduced after PTX; and our results demonstrated the evidence.

London et al reported PAD to be associated with low bone turnover and pronounced osteoblast resistance to PTH in non-diabetic dialysis-dependent ESRD patient [30], which provided a light for possible explanation PTX may be protective for occurrence of PAD. SHPT has been shown to promote cardiac fibrosis and act on endothelial cells to accelerate atherosclerotic processes [28]. It also induced elevation of serum calcium and phosphorus and the metabolic changes play a key role in the pathological process of vascular calcification in intima and medial layer of muscle that increase arterial stiffness and as a marker of inflammatory vascular disease [31, 32]. In an animal study, PTH2 receptor messenger RNA was expressed in the arterial and cardiac endothelium which might support the association between PTH and cardiovascular risk [33]. Previous studies also showed high PTH levels are closely associated with coronary calcification [28, 34], and intima-media thickness of the femoral artery in patients under hemodialysis [35]. Medial arterial calcification might contribute to accelerate PAD in ESRD [36]. PTX in ESRD subjects showed the calcium and PTH level fell dramatically and calcification progression slowed down [18, 37, 38]. This might partially explain our findings.

The curves in Fig 2 presented as a loop-like picture, the 2 curves met after 10 years. The possible explanations involved different pathogenic factors of PAD. Hsu et al. revealed that old age and poor glycemic control to be associated with accelerated progression of PAD in HD patients [39]. Chen et al followed up HD patients for 2 years and found ABI progression was related to high calcium-phosphate product, high fasting glucose and high hs-CRP [40]. Other factors including HD duration and mean blood pressure might promote arterial stiffness and progression off PAD in HD patients [31].

Patients who received PTX in ESRD seemed to have higher income and lower comorbidity like less DM, stroke, and congestive heart failure. It might be related with certain selection bias because those who received PTX might have better physical conditions to receive the operation. The findings of risk factors of PAD in ESRD in our study are quite similar in general population and in previous studies [4, 10, 32].

Though both male and female who have undergone PTX have lower risk of PAD, this effect was more prominent in female subjects. Most of the ESRD patients have multiple comorbidities. As diabetes mellitus and glycemic control are the important predictors for PAD in ESRD patients [28], and in diabetes mellitus and ESRD with PAD, higher amputation rate was observed [41]. Presence of PAD in ESRD indicates poor outcome and higher mortality [41, 42]. According to our findings, female and diabetic patients who developed uncontrolled SHPT might significantly benefit from this treatment. Additional studies might be needed to compare PTX or medical treatment for patients in ESRD with HPT in the development of PAD. Besides, since glycemic control is an important factor to delay the progression of PAD [39, 40], multifactorial intervention might be necessary to prevent the occurrence of PAD or other CV disease to improve the outcomes.

Though we have reported reduced risk of stroke in ESRD patients who received PTX [15], the findings in the present study provide further evidence for the association of controlling SHPT and the risk of PAD. Since PTX was shown to effectively control SHPT in ESRD for 5 years [20], we did not mean to advocate PTX for SHPT in ESRD, but take it as a surrogate to control SHPT. Medical control with either vitamin-D analogues or calcimimetics should be considered first.

There are several limitations in this study. First, the study was conducted based on health insurance claims database; limited by the characteristics of the database, we have no access to the following crucial data: lifestyle factors (such as smoking, BMI and dietary habit), laboratory data (including calcium, phosphate, PTH, Vitamin-D, albumin, and hematocrit… etc.), and certain treatment records (including vitamin-D analogues or calcimimetics). However, also under the NHIRD, we may choose those who had both dialysis-dependent ESRD and had received PTX to ensure the cases of SHPT. Besides, NHI conducted strict claims review for reimbursement to avoid unnecessary surgical operation. With all these conductions together, healthy user bias was minimized. Second, as previously mentioned, there might be selection bias in patients who could afford to receive PTX; we tried to match age, sex or adjusted for comorbidities in both groups to reduce the bias. Third, in non-PTX group, the prevalence of diabetes was higher. Since diabetes is a risk factor for low turnover bone disease [43], it might promote vascular calcifications and clinically significant PAD [44]. However, in our following regression analyses, we adjusted comorbidity to minimize the bias. Fourth, lack of randomization crucially weakened the strength of our research; however, this report shed twilight on the relationship of PAD with PTX in ESRD, and justified further randomized prospective studies in the future.

## Conclusions

PTX is associated with reduced incidence of PAD in dialysis-dependent patients with ESRD. Other significant factors for risk reduction with PTX included female and patients with comorbidity.

## Abbreviations

aHR: adjusted hazard ratio
CI: confidence interval
CKD: chronic kidney disease
DM: diabetes mellitus
ESRD: end-stage renal disease
ICD-9-CM: International Classification of Diseases, Ninth Revision, Clinical Modification
NHIRD: National Health Insurance Research Database
LHID 2000: Longitudinal Health Insurance Database 2000
PAD: peripheral arterial disease
PTX: Parathyroidectomy
