# Correction: The Risk of Peripheral Arterial Disease after Parathyroidectomy in Patients with End-Stage Renal Disease

**DOI:** 10.1371/journal.pone.0158930

**Published:** 2016-07-05

**Authors:** Yueh-Han Hsu, Hui-I Yu, Hsuan-Ju Chen, Tsai-Chung Li, Chih-Cheng Hsu, Chia-Hung Kao

The second author’s name is spelled incorrectly. The correct name is: Hui-I Yu. The correct citation is: Hsu Y-H, Yu H-I, Chen H-J, Li T-C, Hsu C-C, Kao C-H (2016) The Risk of Peripheral Arterial Disease after Parathyroidectomy in Patients with End-Stage Renal Disease. PLoS ONE 11(6): e0156863. doi:10.1371/journal.pone.0156863
